# Integrating indicator-based and event-based surveillance data for risk mapping of West Nile virus, Europe, 2006 to 2021

**DOI:** 10.2807/1560-7917.ES.2024.29.44.2400084

**Published:** 2024-10-31

**Authors:** Kyla Serres, Diana Erazo, Garance Despréaux, María F Vincenti-González, Wim Van Bortel, Elena Arsevska, Simon Dellicour

**Affiliations:** 1 Spatial Epidemiology Lab (SpELL), Université Libre de Bruxelles, Brussels, Belgium; 2 Joint Research Unit Animal, Health, Territories, Risks, Ecosystems (UMR ASTRE), French Agricultural Research Centre for International Development (CIRAD), National Research Institute for Agriculture, Food and Environment (INRAE), Montpellier, France; 3 Unit of Entomology, Department of Biomedical Sciences, Institute of Tropical Medicine, Antwerp, Belgium; 4 Outbreak Research team, Department of Biomedical Sciences, Institute of Tropical Medicine, Antwerp, Belgium; 5 Department of Microbiology, Immunology and Transplantation, Rega Institute, Laboratory for Clinical and Epidemiological Virology, KU Leuven, Leuven, Belgium; * These authors contributed equally to the work and share first authorship.; ** These authors contributed equally to the work and share last authorship.

**Keywords:** epidemic intelligence, risk mapping, ecological niche modelling, event-based surveillance, online news, vector-borne disease, West Nile virus, Europe

## Abstract

**Background:**

West Nile virus (WNV) has an enzootic cycle between birds and mosquitoes, humans being incidental dead-end hosts. Circulation of WNV is an increasing public health threat in Europe. While detection of WNV is notifiable in humans and animals in the European Union, surveillance based on human case numbers presents some limitations, including reporting delays.

**Aim:**

We aimed to perform risk mapping of WNV circulation leading to human infections in Europe by integrating two types of surveillance systems: indicator-based and event-based surveillance.

**Methods:**

For indicator-based surveillance, we used data on human case numbers reported to the European Centre for Disease Prevention and Control (ECDC), and for event-based data, we retrieved information from news articles collected through an automated biosurveillance platform. In addition to these data sources, we also used environmental data to train ecological niche models to map the risk of local WNV circulation leading to human infections.

**Results:**

The ecological niche models based on both types of surveillance data highlighted new areas potentially at risk of WNV infection in humans, particularly in Spain, Italy, France and Greece.

**Conclusion:**

Although event-based surveillance data do not constitute confirmed occurrence records, integrating both indicator-based and event-based surveillance data proved useful. These results underscore the potential for a more proactive and comprehensive strategy in managing the threat of WNV in Europe by combining indicator- and event-based and environmental data for effective surveillance and public health response.

**Key public health message**

**What did you want to address in this study and why?**

West Nile virus is transmitted via mosquitoes to humans and other animals. Infections of West Nile virus are increasing in Europe, and the disease has a considerable impact on human and animal health. We aimed at mapping areas at risk of local circulation of West Nile virus by combining environmental data with notified human cases of West Nile virus infection and data retrieved from news articles.

**What have we learnt from this study?**

By using environmental data and two types of surveillance data, we identified new areas in southern Europe potentially suitable for local circulation of West Nile virus leading to human cases.

**What are the implications of your findings for public health?**

The new areas identified at risk could be used to identify regions requiring targeted enhanced surveillance and preventive measures by public health authorities. Overall, our findings underscore the usefulness of a multifaceted surveillance approach in disease risk mapping.

## Introduction

Emerging and re-emerging vector-borne diseases (VBD) are currently among prime public health concerns in Europe [1]. Over the past years, Europe has seen an increase in the frequency and geographic expansion of VBD, including infections with West Nile virus (WNV), a virus transmitted by mosquitoes of the *Culex* species complex, as well as dengue virus (DENV) and chikungunya virus (CHIKV), transmitted by *Aedes albopictus* and *A. aegypti* mosquitoes [2-4]. The Flavivirus causing WNV infection is maintained in an enzootic bird-mosquito transmission cycle [5,6]. Mosquitoes acquire the infection by feeding on an infected bird and subsequently transmit the virus to mammals, such as humans and horses that only act as dead-end hosts [7,8]. Since the first findings of WNV in Europe in 1959 [9,10], WNV infection has been notified in humans in several European countries, particularly in central and Mediterranean Europe [11].

Climate and land-use changes, increased mobility of people and animals, biodiversity loss and the introduction of non-endemic pathogens and their vectors into new environments have been identified as drivers of spread of vector-borne pathogens [12-14]. Assessing the risk of local circulation of vector-borne pathogens is a critical public health priority. Identifying areas at risk can help health authorities include appropriate epidemiological and entomological investigations, active case finding and vector control activities in their national surveillance plans.

Epidemic intelligence is a framework that encompasses activities related to the early identification, verification, analysis and assessment of potential health threats and investigation to recommend control measures. Epidemic intelligence integrates both indicator-based surveillance (IBS) and event-based surveillance (EBS) components [15]. Indicator-based surveillance (IBS) relies on structured, verified indicators such as reported case numbers collected from routine surveillance systems [16]. In event-based surveillance (EBS), on the other hand, information is collected from various data sources which have not been officially validated by health institutions. Epidemic intelligence was introduced in 2006 as a paramount to more effective disease surveillance, risk assessment and collaboration among European public health institutes [15], and today, a combination of IBS and EBS data are the most common data sources in Europe [16]. Although IBS is the epidemic intelligence keystone, it has limitations, such as potential delays between symptom observation, laboratory confirmation and reporting to the competent authorities, as seen with some European VBD outbreaks [17]. Event-based surveillance has been developed to reinforce traditional surveillance and contribute to early detection of outbreaks and risk assessment of potential health threats, notably by broadening the range of data sources, including informal sources such as news articles and social media reports or information from expert networks such as HealthMap (https://www.healthmap.org/en/), ProMED-mail (https://promedmail.org/) and Platform for Automated extraction of animal Disease Information from the web (PADI-web; https://padi-web.cirad.fr/en/) [18-20].

The French Epidemic Intelligence System (FEIS, also known in French as Veille Sanitaire Internationale) created PADI-web [18], an automated biosurveillance system, designed to gather, process and extract epidemiological information from online news sources [21]. This platform collects news articles using custom Really Simple Syndication (RSS) feeds. These feeds search for articles by linking terms related to the disease or syndrome of interest, filtering out extraneous elements like ads, images and comments [18,21]. Furthermore, machine-learning techniques are implemented in PADI-web to identify the pertinent epidemiological features in the text (e.g. disease, affected host, clinical signs, outbreak location coordinates, publication date). Currently, PADI-web collects disease information from Google News due to its global coverage and support for multiple languages.

Many studies on risk mapping of VBD in Europe rely on data collected through an IBS approach. For instance, Salami and colleagues used machine learning and model-agnostic methods to predict the importation risk of DENV into Europe using monthly data on imported cases of dengue from the European Centre for Disease Prevention and Control (ECDC) [22]. Watts and colleagues used data on human cases of WNV infection reported to ECDC and climate, land-use and economic-data to identify risk factors associated with WNV infection in southern and south-eastern Europe [23]. Farooq and colleagues applied a supervised machine learning approach to map the WNV outbreak risk in Europe using an ensemble climate model with ECDC human case data [24].

In this study, we assessed how EBS data could complement traditional IBS disease data to enhance risk mapping of VBDs in Europe, using WNV as a case study. While ECDC also uses EBS to monitor global public health threats in Europe [25], its use of EBS differs from our approach, as we focus on applying EBS specifically for risk mapping. In our study, we used both EBS and IBS data to train ecological niche models using a boosted regression trees (BRT) approach to estimate the ecological suitability of WNV given local environmental conditions. These analyses allowed us to investigate the added value of using the integrated IBS and EBS datasets for risk mapping of local WNV circulation leading to human infection.

## Methods

### Data on West Nile virus infection in humans

The IBS data used in our study were retrieved from the European Surveillance System (TESSy) of the ECDC which provided us aggregated data on confirmed human cases of WNV infection from August 2006 to November 2021 at the NUTS (nomenclature of territorial units for statistics) level 3 (NUTS3) [26]. We curated the dataset to include only autochthonous cases, defined as infections of local origin, i.e. not associated with a recent travel-history. We retrieved the EBS data on WNV infections in humans from January 2006 to December 2021 from the PADI-web platform. In April 2022, we collected news reports identifying outbreak signals of WNV infections up to December 2021.

**Figure 1:**
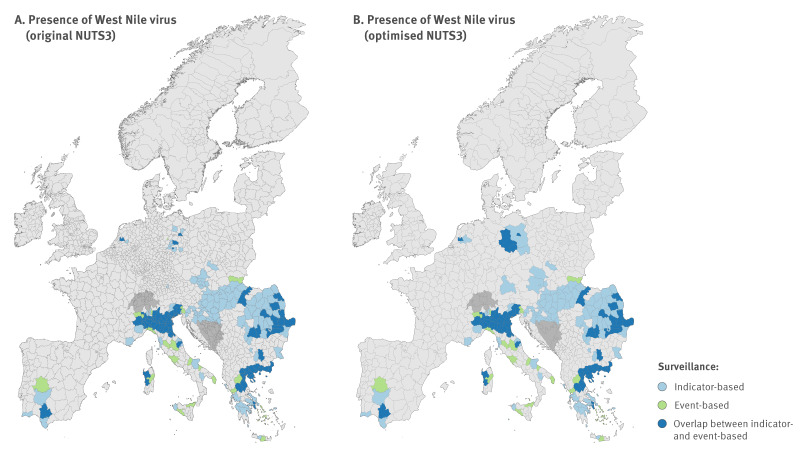
Confirmed autochthonous human cases of West Nile virus infection, Europe, 2006–2021

NUTS3: nomenclature of territorial units for statistics, level 3; WNV: West Nile virus.

We defined an outbreak as a location associated with at least one autochthonous case. We set feeds using the keyword ‘West Nile virus’ and translated the keyword by AGROVOC Multilingual Thesaurus (https://www.fao.org/agrovoc/) to 16 languages spoken in European countries (Austria, Bulgaria, Croatia, Czechia, France, Germany, Greece, Hungary, Italy, the Netherlands, Portugal, Romania, Serbia, Slovakia, Slovenia and Spain) where WNV cases had been previously documented in the WNV epidemiological update of ECDC [27]. We then systematically verified all the news articles identified by PADI-web and only retained those reporting at least one autochthonous human case of WNV infection in Europe. We manually verified that the outbreak event coordinates identified by PADI-web corresponded to outbreak sites mentioned in the news article. We excluded the following entries: duplicated locations (i.e. previously identified outbreak location), country-level locations, locations not associated with an outbreak, locations associated with other disease cases and words mistakenly identified as outbreak locations by the system pipeline. Furthermore, any missing outbreak locations not automatically identified by the system pipeline were added by finding the coordinates through Google Earth. The resulting dataset was considered a moderator-verified dataset of WNV occurrence records. These locations were not cross-checked with public health authorities or scientific literature to confirm whether they corresponded to confirmed cases. To ensure consistency in the geographical granularity of the IBS records, we used the R package (R Foundation, Vienna, Austria) ‘gisco’ version 0.3.5 (https://zenodo.org/doi/10.5281/zenodo.4317946) [28] to match each EBS case or outbreak coordinates to the associated NUTS3 level (Figure 1).

Panel A. Original NUTS3 areas are displayed.

Panel B. Optimised NUTS3 used in the ecological niche modelling analyses are displayed.

Areas with at least one confirmed autochthonous case of WNV infection in humans in the indicator-based surveillance are shown in light blue; areas with at least one autochthonous case of WNV infection in humans detected in the event-based surveillance are shown in light green; areas with autochthonous cases of WNV infection in humans detected in both indicator and event-based surveillance are shown in dark blue. Darker grey areas correspond to Switzerland and Bosnia and Herzegovina, countries that are not included in the European Centre for Disease Prevention and Control surveillance of West Nile virus infection.

At the end, we compiled two datasets: one with IBS data and another with IBS and EBS data, EBS data verified by moderators. Based on these datasets, we subsequently categorised NUTS3 areas as presence or absence sites for WNV infections in humans. Presence was categorised as areas with at least one confirmed or verified autochthonous human case of WNV infection and absences as areas where no autochthonous human cases of WNV infection were observed (Figure 1).

### Environmental data

We used 14 environmental factors documented to influence the risk of circulation of WNV in Europe. These environmental factors were selected from a literature review by Giesen and colleagues [29]: human population density, air temperature (by season), precipitation (by season), relative humidity (by season), soil moisture, leaf index (plant canopies), urban areas, wet crops, pastures, agroforestry areas, forest areas, arable lands, arid shrublands, wetlands and open water areas. Summer covered June–August, autumn September–November, winter December–February and spring March–May. These environmental variables were retrieved from three databases, listed in Supplementary Table S1: (i) the Inter-Sectoral Impact Model Intercomparison Project (ISIMIP), which focuses on quantifying climate-related risks under various climate change and socioeconomic conditions, (ii) ERA5, the fifth generation of the European Centre for Medium-range Weather Forecasts (ECMWF) reanalysis, offering comprehensive global climate and weather information and (iii) Corine Land Cover, a programme led by the European Commission that provides pan-European land cover data classified into 44 categories.

### Optimisation of the map of administrative areas

The European Union (EU) has developed NUTS, the regional classification which mirrors the territorial administrative divisions of European countries [26]. The NUTS divides each EU country into three levels, based on population size in the region. At the third administrative level, the sizes of the NUTS (NUTS3) areas can considerably vary across European countries. For instance, NUTS3 areas in Germany are relatively small and comparable in size to NUTS level 4 areas in some other countries. In our ecological modelling analyses, we created an optimised NUTS map, i.e. a map where the NUTS areas were as uniform in polygon size as possible. This optimised GIS shapefile was specifically crafted for the European network for medical and veterinary entomology (VectorNet) [30], a project co-led by the ECDC and the European Food Safety Authority (EFSA).

### Ecological niche modelling

To map the ecological suitability of Europe for WNV circulation leading to human cases, we implemented a BRT approach [31]. This machine learning algorithm generates a collection of sequentially fitted regression trees that optimise the predictive probability of occurrence given local environmental conditions. In this study, such a predictive probability can be seen as an indication of how suitable an environment is, ranging from 0 to 1, with higher values indicating a greater degree of ecological suitability for local WNV circulation. The BRT approach is able to estimate nonlinear relationships between the response and predictive variables, even in the presence of multicollinearity [31]. However, the algorithm can be computationally expensive and prone to overfitting if not properly regularised [32]. We used the BRT algorithm implemented in the R package ‘dismo’ version 1.3.9 (https://cran.r-project.org/web/packages/dismo/index.html).

To investigate the usefulness in integrating EBS data for modelling the risk of local WNV circulation, we trained two ecological niche models: one based on IBS data and the other on IBS and EBS data. Specifically, we implemented a Bernoulli BRT approach on the presence and absence data, running 100 independent replicate BRT analyses of both datasets to ensure robustness and accuracy. The 100 resulting maps of ecological suitability were eventually averaged for visualisation purposes. The optimised NUTS3 areas with at least one confirmed autochthonous case were considered presence locations, the others as potential pseudo-absence locations. We chose to analyse presence and pseudo-absence data rather than incidence data to avoid treating the absolute number of cases as a reliable proxy for prevalence of WNV infection [33].

To investigate the robustness and sensitivity of our results to the sampling intensity of pseudo-absences across the study area, we further trained ecological niche models based on alternative subsets of potential pseudo-absences. While the main analyses were based on pseudo-absences sampled across 50% of the administrative areas where no WNV presence had been confirmed, we also considered alternative datasets of pseudo-absences sampled from 25%, 75% and 100% of the administrative areas not associated with a presence record. In each case, we re-trained 100 ecological niche models. Based on these newly generated models, we then re-evaluated the difference in ecological suitability between models incorporating both IBS and EBS data and those considering only IBS data.

To address spatial autocorrelation and prevent model overfitting, we opted for a spatial instead of the standard cross-validation procedure because the standard one often overestimates the predictive ability of the model when occurrence data exhibit spatial autocorrelation [34]. Specifically, we applied the spatial cross-validation procedure based on block generation introduced by Valavi and colleagues, implemented in the R package ‘blockCV’ version 3.0.0 [35]: our presences/pseudo-absences dataset was split into five spatial folds determined by this block generation method. We trained the BRT models using the following parameter values: a tree complexity of 5, a learning rate of 0.005 and a step size of 10.

The predictive performance of each BRT model was calculated by computing the area under the receiver operating characteristic curve (AUC). Values of AUC close to 1 indicate high model predictive performance, while AUC values close to or below 0.5 indicate poor predictive performance, presented in Supplementary Table S2. Because the AUC metric has been criticised in previous studies due to its dependence on prevalence [36-39], we also estimated the predictive performance of the model by calculating the prevalence-pseudoabsence-calibrated Sørensen’s index (SI_ppc_) [40,41], which has a minimum value of 0 and a maximum value of 1 representing the highest predictive performance, data presented in Supplementary Table S2. While our ecological suitability estimates ranged between 0 and 1, the computation of this index requires binary presence-absence estimates. For the SI_ppc_ computations, we thus conducted an optimisation process that involved varying threshold values from 0 to 1 in increments of 0.01 to select the threshold value maximising the SI_ppc_ [39,42].

To determine the contribution of each environmental variable, we calculated their relative influence (RI) in the BRT models, as presented in Supplementary Table S3. For a specific environmental factor, the RI value is computed as the number of times this factor is selected for splitting a tree, weighted by the squared improvement to the model resulting from each split and averaged over all trees [31]. Additionally, partial dependence plots or response curves were plotted to show how ecological suitability varies with one specific predictor variable while all others are kept at a constant mean, presented in Supplementary Figure S1. This visualisation technique enhances our understanding of the model behaviour and aids in interpreting the relationships between the different predictors and the response variable [31].

**Figure 2:**
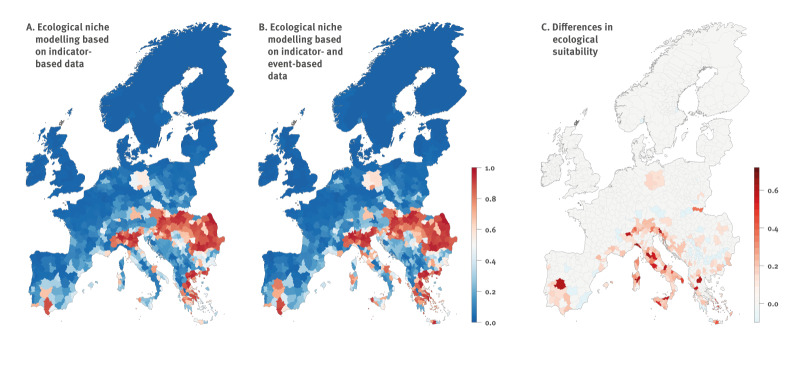
Comparison of West Nile virus risk maps obtained when analysing indicator-based surveillance data and indicator-based and event-based surveillance data, Europe, 2006–2021

EBS: event-based surveillance; ENM: ecological niche modelling; IBS: indicator-based surveillance; NUTS: nomenclature of territorial units for statistics.

Finally, we evaluated the changes in ecological suitability between the two averaged ecological suitability maps obtained when solely analysing the IBS data and when analysing both IBS and EBS data. This involved subtracting the estimated ecological suitability values of each optimised NUTS3 area with the model trained on both IBS and EBS data from those estimated for the same area with the model only trained on IBS data (Figure 2C). Additionally, we calculated the corresponding area at risk by determining the area of each NUTS3 area associated with an ecological suitability value that increased by 5%, 10% and 20%, respectively.

Panel A. The map shows the ecological suitability of optimised NUTS level 3 areas for local circulation of West Nile virus, as estimated by ENM analyses based on IBS data.

Panel B. The map shows the ecological suitability of optimised NUTS level 3 areas for local circulation of West Nile virus, as estimated by ENM analyses based on a combination of IBS and EBS data. These maps display the averaged ecological suitability values estimated by 100 replicates of the boosted regression tree analysis.

Panel C. The map highlights the difference in ecological suitability values. Areas coloured in red indicate areas with higher ecological suitability estimates when the model is trained on the combination of IBS and EBS data, while light blue areas represent areas with higher ecological suitability estimates when the models are only trained on IBS data.

## Results

From 2006 to 2021, autochthonous human cases of WNV infection with known places of infection were reported to ECDC in 182 NUTS3 administrative areas from 16 countries [43]. In addition, PADI-web detected 706 articles, of which 250 met our inclusion criteria. From these, the algorithm automatically identified 2,356 locations associated with outbreaks of WNV infections in humans. After manual verification, 766 locations were confirmed as unique outbreak sites. We excluded 600 country-level locations, 650 duplicates, 270 unrelated to outbreaks of WNV infection and 70 associated with other VBD. Additionally, we manually added 192 locations missed by the algorithm. In total, 958 locations were included, corresponding to 92 NUTS3 areas identified by PADI-web, of which 22 were not associated with any IBS record (Figure 1).

All our trained ecological niche models showed good predictive performance as measured by the AUC and SI_ppc_ metrics, displaying values > 0.8, as presented in Supplementary Table S2. In our study, climatic factors, namely seasonal temperature, relative humidity and precipitation, exhibited the highest RI (> 5%) in both kinds of models, i.e. either trained on IBS or IBS and EBS data, as presented in Supplementary Table S3 and Supplementary Figure S1. Specifically, summer air temperature (RI IBS = 14.99%; RI IBS + EBS = 17.93%), summer precipitation (RI IBS = 8.73%; RI IBS + EBS = 8.64%), spring relative humidity (RI IBS = 13.87%; RI IBS + EBS = 5.42%) and autumn relative humidity (RI IBS = 6.64%; RI IBS + EBS = 6.55%) contributed significantly to both kinds of models. Winter precipitation (RI IBS = 6.02%; RI IBS + EBS = 7.59%) and winter relative humidity (RI IBS = 15.34%; RI IBS + EBS = 20.25%) also displayed notable RI values. Furthermore, our response curves show that temperatures ranging from 15 to 28°C, precipitation from 0.2 to 0.5 cm and relative humidity levels from 70 to 85% had a positive influence on the probability of the occurrence of human cases of WNV infection, as presented in Supplementary Figure S1. As demonstrated in the response curves in Supplementary Figure S1, both kinds of models also showed clear positive associations between ecological suitability for local WNV circulation and human population density, urban areas, open water areas, wet crops and wetlands, as well as the level of soil moisture. The relative influence of these variables was generally similar between both models, with a difference of < 1%, except for open water areas (1.4% difference). Specifically, the BRT models trained on integrated IBS and EBS data assigned a higher RI to open water, making it the top variable among the land-use variables, as shown in Supplementary Table S3. Regarding the other land-use variables, arable land, leaf index and forest areas were negatively associated with the ecological suitability for local WNV circulation, as presented in Supplementary Figure S1.

As shown in Figure 2, we compared the ecological suitability estimated for each NUTS3 area when using IBS data (Figure 2A) or a combination of IBS and EBS data (Figure 2B). Both kinds of models identified highly suitable regions for local WNV circulation leading to human cases throughout southern Europe, particularly in southern Iberian Peninsula, southern France, Italy, the Balkan Peninsula and countries in the Danube River Basin. The models trained on both IBS and EBS data identified additional areas potentially at risk of local WNV circulation, specifically in central and southern Italy, southern Spain, southern France and Greece (Figures 2B,C). As detailed in Supplementary Figure S2, these inferred trends remained consistent with the results obtained when training the ecological niche models considering all or alternative subsets of potential pseudo-absences.

To further quantify these predictive differences between the models trained on IBS or IBS and EBS data, we computed the total area of the NUTS3 associated with an ecological suitability value that had increased by 5%, 10% and 20% when considering both IBS and EBS data. Among the NUTS3 areas with greater ecological suitability (red areas in Figure 2C), 151 demonstrated an increase of ≥ 5% (corresponding to a total area of 587,766 km^2^), 78 an increase of ≥ 10% (corresponding to a total area of 307,200 km^2^) and 31 an increase of ≥ 20% (corresponding to a total area of 114,010 km^2^). Additionally, we identified 47 NUTS3 areas exhibiting higher ecological suitability when solely training the models on IBS data (light blue areas in Figure 2C). Among the 151 NUTS3 areas with a difference of ≥ 5% or more in ecological suitability, 41 corresponded to WNV presences identified by both IBS and EBS data, while 21 solely corresponded to EBS data, totalling 62 NUTS3 areas with WNV occurrence records.

## Discussion

The spatial distribution, number and size of outbreaks of WNV infection in humans vary across the European continent, underlying the complexity of WNV ecology and its increasing significance for public and animal health. Implementing effective disease surveillance and control strategies are of high importance. Our study showcases the use of a non-traditional surveillance tool to enhance disease risk mapping.

In integrating EBS data from PADI-web into an IBS dataset, we assessed the added value of incorporating news sources to detect additional areas at potential risk of WNV circulation. All our ecological niche models identified ecologically suitable areas in the southern European countries, corroborating previous studies [2,11,27,44,45]. The models integrating EBS data highlighted additional potentially ecologically suitable areas for local WNV circulation, specifically in southern Spain, southern France, central/southern Italy and Greece, which aligns with findings by García-Carrasco and colleagues [46].

Our analyses confirmed that several key environmental factors favour local WNV circulation, particularly climate variables, which contributed most to our ecological niche models. Consistent with prior research, we found that temperatures between 15 and 28°C seem to be important in determining the risk of local WNV circulation. This aligns with studies indicating that temperatures varying in the range of 15 and 28°C favour mosquito development, survival and extrinsic viral replication [47,48]. Additionally, precipitation and relative humidity are also important in the mosquito life cycle and thus in WNV transmission. Increased precipitation can lead to the formation of mosquito larval habitats [49], while high humidity has been linked to increased egg production and hatching, thereby increasing mosquito abundance [49]. Furthermore, winter rainfall is positively correlated with mosquito abundance in the temperate Mediterranean regions [50]. Regarding land-use variables, we identified wetlands, wet crops and open water areas as important contributors to our models. In fact, these are known to act as bird nesting grounds, creating optimal conditions for establishing WNV enzootic cycles [50]. Conversely, urban areas, along with higher human population density, elevate WNV transmission risk by facilitating increased encounters between hosts and infected vectors. Human activities, such as storm water sewage systems in these areas contribute to increased mosquito abundance by creating breeding sites [51].

Our modelling process does present certain limitations. Firstly, it does not incorporate biotic factors like bird diversity, abundance or migratory pathways which could offer insights into the ecological suitability of WNV. Additionally, we chose not to include animal cases, as the differences in surveillance of WNV in animals may vary more than surveillance of WNV infection in humans between European countries. To prevent circulatory issues, we also did not consider available *Culex* mosquito species distribution models trained on similar sets of environmental variables.

In addition, we acknowledge a series of limitations inherent to collecting and curating EBS data. We noticed that PADI-web algorithm prioritises the most recent news articles, especially in cases where topics, such as WNV outbreaks, receive considerable media attention. Therefore, as our study queries were implemented in 2021, the algorithm exhibited a preference for identifying recent WNV outbreaks (i.e. WNV outbreaks occurring during the last 5 years from our PADI-web search). This may explain why we retrieved few articles for the period 2006–2016. Moreover, variations in news coverage on VBD between countries constitute another potential limitation. Lastly, while EBS epidemiological data were automatically retrieved from EBS platforms, they required moderator screening and verification due to the current limitations of machine learning algorithms compared with human expertise. The verification step, therefore, requires a considerable amount of time, which can vary depending on the volume of records and the number of human moderators. This is a recognised limitation among epidemic intelligence users across surveillance levels, from global entities such as the World Organisation for Animal Health (WOAH) and the World Health Organization (WHO) to regional bodies like the ECDC, to national platforms such as the Plateforme Nationale d’Épidémiosurveillance en Santé Animale in France. Nonetheless, there is a growing recognition of the need for machine support, particularly in automatically extracting epidemiological information. To address this, we are enhancing the existing PADI-web algorithm for spatial information extraction to improve the precision of automatically retrieved epidemiological information, such as geographical coordinates corresponding to outbreak locations. This enhancement aims to expedite the verification step, thereby reducing the time required for curation. Despite the time investment, using EBS data was valuable for detection of 22 at-risk locations of WNV not captured by IBS alone at a NUTS3 area of precision. It is crucial for countries to prioritise the precise reporting of cases to public health authorities, as this significantly impacts the accuracy of model results. While challenges persist, EBS presents an avenue for enhancing occurrence datasets and improving disease surveillance efforts. Of note, as EBS data undergo moderator verification and are considered unofficial data, they must not be misconstrued as validated occurrence data.

Along with the recent work of Fanelli and colleagues exploiting EBS data for mapping the risk of Crimean-Congo haemorrhagic fever [52], our study is one of the latest explorations of integrating EBS data in risk mapping of a VBD. While they do not constitute official or confirmed infection cases, such complementary EBS data may provide valuable information in areas that might already have local circulation not yet detected by the official surveillance systems. The EBS data could enrich IBS datasets by revealing additional locations with cases, enhancing geographic granularity and potentially bridging potential surveillance gaps [53]. Areas associated with an increased estimated risk when both EBS and IBS data were included to train ecological niche models could constitute new areas for closer monitoring of local virus circulation and where preventive measures could be implemented by public health authorities. Overall, incorporating EBS data in disease risk mapping assessment has the potential of improving surveillance and subsequent design of control measures.

## Conclusion

By highlighting new areas potentially at risk of local circulation of WNV, our study illustrated the benefits of integrating EBS data along with IBS data. Event-based data could be an important tool for automatic and tailored health data collection, offering a dynamic and complementary approach for monitoring health threats. Finally, EBS could prove particularly advantageous in regions where the target pathogen is not yet known to circulate. In these areas, existing surveillance systems may be less established, and integrating EBS data can help address surveillance bias issues until adequate surveillance systems are implemented.

## Disclaimer

The views and opinions of the authors expressed herein do not necessarily state or reflect those of ECDC. The accuracy of the authors’ statistical analysis and the findings they report are not the responsibility of ECDC. ECDC is not responsible for conclusions or opinions drawn from the data provided. ECDC is not responsible for the correctness of the data or data management, data merging, and data collation after the provision of the data. ECDC shall not be held liable for improper or incorrect use of the data.

## Supplementary Data

27000 Supplementary Material 1

## Supplementary Data

91000 Supplementary Material 2

## Supplementary Data

24000 Supplementary Material 3

## Supplementary Data

3139000 Supplementary Material 4
