# Supplementary material for: Integrating indicator-based and event-based surveillance data for risk mapping of West Nile virus, Europe, 2006 to 2021
Source: Euro Surveill. 2024 Oct 31;29(44):2400084. doi: 10.2807/1560-7917.ES.2024.29.44.2400084 (PMC11528904; doi:10.2807/1560-7917.ES.2024.29.44.2400084)
Supplement: Supplementary Material 4 [file 24-00084_SERRES_SupplementaryFigures.pdf]

This supplementary material is hosted by *Eurosurveillance* as supporting information alongside the article "Integrating indicator-based and event-based surveillance data for risk mapping of West Nile virus, Europe, 2006 to 2021", on behalf of the authors, who remain responsible for the accuracy and appropriateness of the content. The same standards for ethics, copyright, attributions and permissions as for the article apply. Supplements are not edited by *Eurosurveillance* and the journal is not responsible for the maintenance of any links or email addresses provided therein.

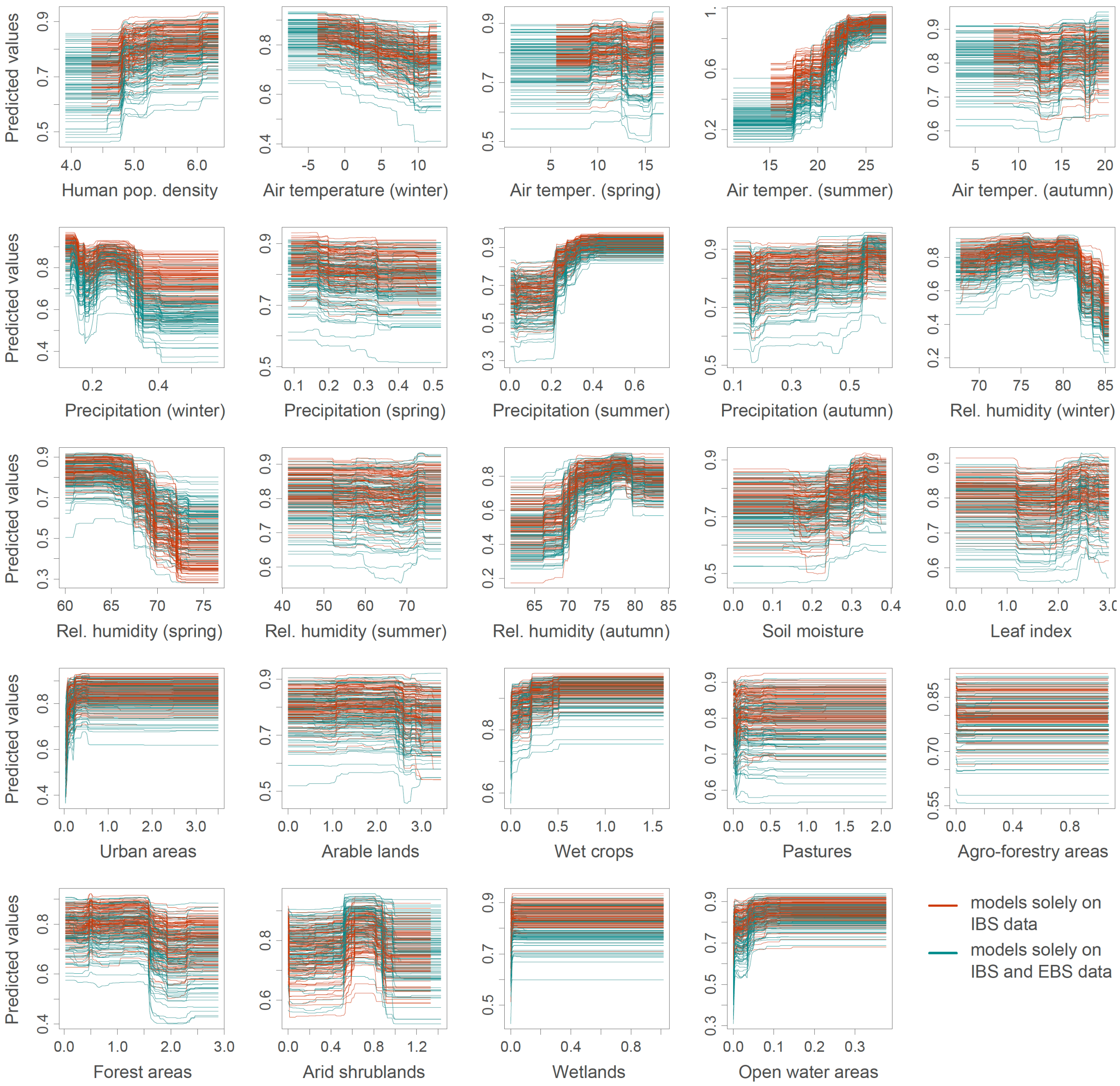

Figure S1. Response curves of the ecological niche models. For each environmental factor, we report the response curve of each of the 100 replicate boosted regression tree (BRT) models trained while considering either indicator-based surveillance (IBS) data (red curves) or a combination of IBS and event-based surveillance (EBS) human infection records (blue curves). These graphs describe the relationship between the environmental values (x-axes) and the response (y-axes), i.e. the ecological suitability of WNV, and were obtained by computing the ecological suitability variation associated with one specific variable, while all others were kept constant at their median value. The following environmental variables were analysed: human population density (log_{10}-transformed), air temperature by season (in Celsius degrees), total precipitation by season (in cm), relative humidity by season (%), soil moisture (in m^3), leaf index (%), urban areas (%), arable lands (%), wet crops (%), pastures (%), agro-forestry areas (%), forest areas (%), arid shrublands (%), wetlands (%), and open water areas (%).

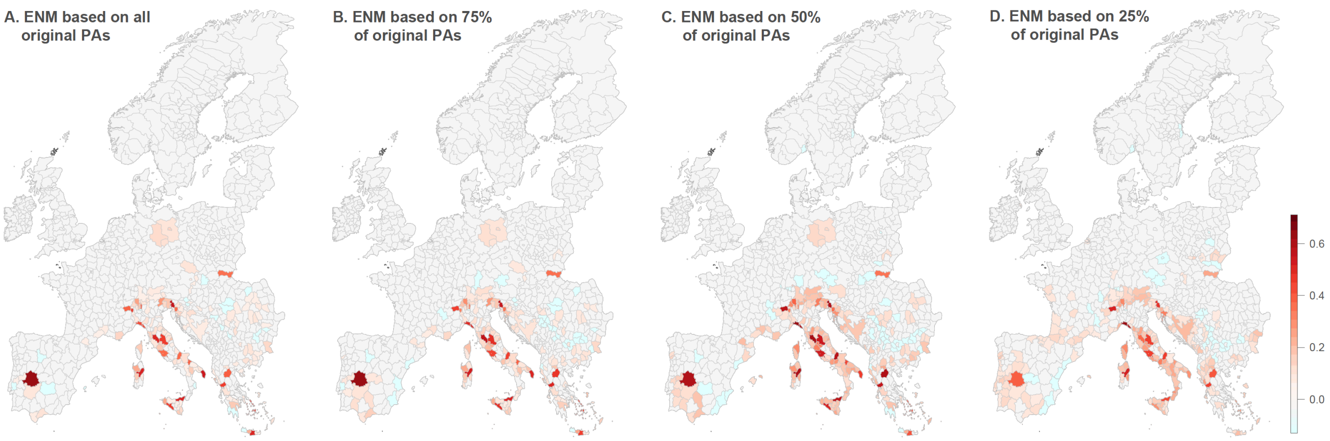

Figure S2. Investigations of the robustness and sensitivity of our ecological niche models to the sampling intensity of pseudo-absences across the study area. These maps highlight the differences between the averaged ecological suitability values estimated when considering both IBS and EBS data and when only considering IBS data. While the first map (A) is obtained when training ecological niche models with pseudo-absences corresponding to all optimised NUTS3 administrative areas with zero confirmed non-imported human cases of WNV infection, the three other maps (B-D) are obtained when training ecological niche models on random subsets of all available pseudo-absences. Specifically, we considered both thresholds of 75% (B), 50% (C; corresponding to Figure 2C) and 25% (D) of all pseudo-absence data and, for each threshold, we re-trained 100 ecological niche models each time based on a random subset of pseudo-absence data.
